# Determinants of Test Variability in Scotopic Microperimetry: Effects of Dark Adaptation and Test Indices

**DOI:** 10.1167/tvst.10.1.26

**Published:** 2021-01-15

**Authors:** Giovanni Montesano, Timos K. Naska, Bethany E. Higgins, David M. Wright, Ruth E. Hogg, David P. Crabb

**Affiliations:** 1 Optometry and Visual Sciences, City, University of London, London, UK; 2 NIHR Biomedical Research Centre, Moorfields Eye Hospital NHS Foundation Trust and UCL Institute of Ophthalmology, London, UK; 3 Centre for Public Health, Queen's University Belfast, Royal Hospital, Belfast, Northern Ireland

**Keywords:** microperimetry, scotopic sensitivity, dark adaptation, test–retest variability

## Abstract

**Purpose:**

To test the effect of different dark adaptation conditions and reliability indices on the variability of two color scotopic microperimetry.

**Methods:**

We analyzed data from 22 consecutive visually healthy adults. Scotopic microperimetry was performed (Macular Integrity Assessment microperimeter, CenterVue, Padua, Italy) with two wavelength stimuli, cyan (505 nm) and red (627 nm), after a dark adaptation time of 10, 20, or 30 minutes. All tests were repeated twice to measure test–retest variability with Bland–Altman plots. We also provide a method to more accurately quantify the false-positive (FP) responses based on response data (button pressing) from the device, similar to FP responses used in standard static perimetry. Data on fixation stability (95% bivariate contour ellipse area) and blind spot responses were also extracted. Their relationship with measured sensitivity (in decibels) and test–retest variability was quantified through linear mixed effect models.

**Results:**

Dark adaptation had a significant effect on the sensitivity (dB) measured with the cyan stimulus (*P* < 0.001), but no effect on the red stimulus. Of the three metrics, the novel FP responses showed the best association with test–retest variability and was the only predictor consistently significant for all tests (*P* < 0.01).

**Conclusions:**

Dark adaptation protocols should be carefully standardized for scotopic testing, especially if a cyan stimulus is used. The proposed FP responses should be used to assess reliability of microperimetry examinations instead of other metrics.

**Translational Relevance:**

We developed a method to calculate a more accurate estimate of the FP responses using data available to all researchers, generalizable to all Macular Integrity Assessment microperimeter tests.

## Introduction

In recent years, perimetric tests have been acknowledged to be a useful tool for investigating macular disorders such as age-related macular degeneration (AMD). However, people with macular damage may not be able to reliably fixate on a central target throughout a test,^1^ a requirement in standard automated perimetry. This factor could limit the spatial accuracy and reliability of the test. Microperimetry compensates for eye movements via real-time retinal tracking through infrared imaging, providing spatially registered sensitivity measurements mapped onto a fundus image of the retina.^2^^–^^4^ Being a functional measure, microperimetry is an approved end point for clinical trials by the Food and Drug Administration.^5^ The Macular Integrity Assessment microperimeter (MAIA, CentreVue, Padova, Italy) is one of the most commonly used microperimetry devices. The MAIA uses a mesopic background (1.27 cd/m^2^) and tests the retinal sensitivity through the presentation of white stimuli (Goldmann III size).^6^ People with early and intermediate AMD report worse visual function under dimly lit conditions. Hence, scotopic-based investigation of visual function is particularly pertinent in AMD research.^7^^,^^8^ A modified version of the MAIA, the Scotopic MAIA (S-MAIA, CentreVue), allows for the investigation of retinal sensitivity under dark-adapted scotopic conditions.^9^ Moreover, the retinal sensitivity can be tested with monochromatic stimuli with two different wavelengths, cyan (505 nm) and red (627 nm), which are thought to preferentially probe rod-mediated and cone-mediated function, respectively.^10^^–^^12^

Rod latency increases with the length of dark adaptation during scotopic investigations. Therefore, it is expected that the length of the dark adaptation protocol would impact rod-mediated function, as assessed by the response to the cyan stimulus.^9^^–^^12^ The effect of dark adaptation has been recently explored for mesopic microperimetry,^13^ but not for the scotopic examination. This point is important, because inconsistent dark adaptation protocols might affect test–retest variability because they would introduce systematic differences between test repetitions and yield inaccurate results.

We aimed to examine the effect of different dark adaptation protocols on the microperimetric test performed with the S-MAIA in terms of recorded sensitivity and test–retest variability in visually healthy people. Test–retest sessions with three different adaptation protocols were conducted for both types of scotopic stimuli (red and cyan). This data collection also offers a precious opportunity to explore the effect of other test parameters on test–retest variability in microperimetry, such as fixation instability and false-positive (FP) responses. Importantly, a metric to explicitly estimate FP is not yet available in microperimetry and these are usually inferred from indirect analysis of blind spot responses (BSR). We provide a method to calculate a more reliable estimate of FP errors based on test information provided by the machine and easily accessible to researchers. We show that this is the main determinant of test–retest variability among all indices and that it should be used instead of the currently available metrics.

## Methods

### Testing Procedure

The study had Institutional Review Board approval from the research ethics committee Queens University Belfast, School of Medicine Dentistry & Biomedical Sciences (Ref.16.37v3) and adhered to the tenets of the Declaration of Helsinki. Twenty-four visually healthy adults (>18 years of age) were recruited for this study. Exclusion criteria included diagnosis of any ocular disease, opaque ocular media, high refractive error (±10 diopters) and a history of squint. Only one eye per subject was tested. The eye with less refractive error was selected after an autorefraction measurement (ACCUREF K-900, Shin-Nippon, Japan). The microperimetric tests were performed with a S-MAIA in scotopic conditions. Each test was composed of two sessions where the same locations were tested with stimuli of two different wavelengths, 627 nm (red stimulus) and 505 nm (cyan stimulus). The testing protocol was preceded by a training session with a fast examination, to familiarize all subjects with the test. Each subject was tested after three different dark adaptation sessions lasting 10 minutes, 20 minutes, and 30 minutes (0.001 lux ambient light). Each session was followed by a 10-minute break in mesopic conditions (600 lux). The same testing sequence was repeated a second time in a separate visit (retest), excluding the training examination. The minimum interval between visits was 1 day and the maximum was 7 days. As per manufacturer's default settings, for each session, the test with the cyan stimulus was always performed first, followed by the test with the red stimulus at the same locations.

**Figure 1.**
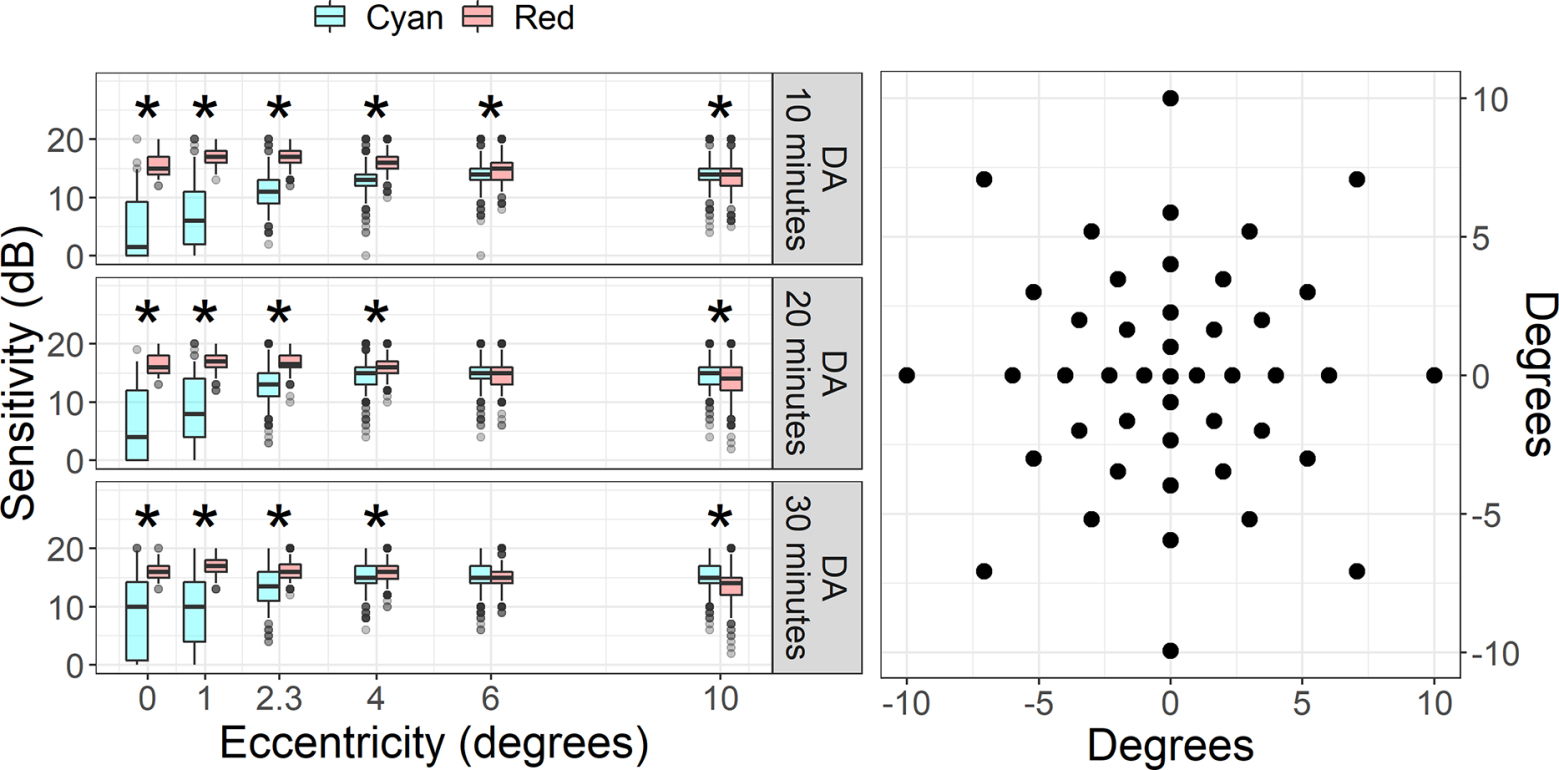
On the left, box plots representing the sensitivity at each eccentricity for tests performed with different dark adaptation protocols. The boxes enclose the 25th to 75th percentiles, the whiskers extend from the 5th to the 95th percentiles. Asterisks indicate the eccentricities for which the difference between the red and cyan stimulus was significant (*P* < 0.05, Bonferroni–Holm correction for six tests). On the right, a schematic of the grid locations used for testing.

The microperimeter uses continuous infrared imaging to track and compensate for eye movements during the test.^2^^–^^4^ All tests start with a short period during which fixation is monitored without any stimuli except for the central target to determine the preferred retinal locus (PRL) of fixation on the retina; this is then used as the center of the testing grid. In this experiment, a new PRL was determined at the beginning of each test (see the Supplementary Material for a detailed analysis of the effect of changes in PRL positions). Tested locations were positioned along concentric rings at 1.0°, 2.3°, 4.0°, 6.0°, and 10.0° from fixation (Fig. 1). Sensitivity is determined using a standard 2–1 staircase using Goldman III size stimuli (0.43 degrees in diameter). It is important to acknowledge that the implementation of the S-MAIA used for this experiment was an early version with a limited dynamic range (20 dB). The range of stimulus intensity corresponds with 10 to 30 dB in the newest version of the S-MAIA, which has extended both the lower and the upper dynamic range. The whole dynamic range is now 36 dB. For consistency with our data, we report the sensitivity values as they were extracted from the XML file (discussed elsewhere in this article), ranging from 0 to 20 dB.

### Test Metrics

Point-wise sensitivity values were extracted from the XML files provided by the machine. The average test sensitivity is calculated as the mean of all the values in a test, excluding the blind spot, and is extracted from the XML. We also extracted the 95% bivariate contour ellipse area (BCEA) (deg^2^), the BSR, the duration of the test (in seconds) and the average response time (ART, in milliseconds). The BCEA is a measure of fixation stability and represents the area of the ellipse enclosing 95% of the fixation positions observed during the test. Large BCEA values indicate an unstable fixation. The BSR is the percentage of times the subject responded when a stimulus was projected on the site of the blind spot, corresponding with the location of the optic nerve head; this entity is detected manually by the operator on the infrared fundus picture at the beginning of the test. Because eye movements are compensated by the fundus tracking, this can be interpreted as a surrogate metric of FP results. However, blind spot tests are performed with relatively bright stimuli (10 dB) and can be easily affected by wrong positioning of the optic nerve head landmark or produce stray light seen by the subject. Therefore, we devised a different metric that would effectively measure the rate of FP response using data within the XML file. This file reports so-called wrong pressure events, that is, the number of times the subject pressed the response button outside the response windows, a fixed period of time (1500 ms) after the stimulus has been presented. We denote these to be wrong responses. Using this value, we can calculate the rate of FP responses knowing the total time during the test when no true responses were expected. The details of the computation are reported in the Appendix.

### Statistical Analysis

We used mixed effect models to compare the perimetric sensitivity (dB) at different eccentricities between the cyan and the red stimulus. The random effect was a random intercept for the subject. The fixed effects were the type of stimulus, the dark adaptation condition, and the eccentricity as a discrete factor, including their interaction. *P* values were corrected for multiple comparisons (six for each dark adaptation condition) using the Bonferroni–Holm method. A similar model, excluding eccentricity, was used to evaluate the average differences in the ART and BCEA between the two stimuli and between dark adaptation conditions. The effect of different metrics on the measured sensitivity was also measured using mixed effect models, with a random intercept term for the subjects. Each model was fitted twice, that is, separately for the red and cyan tests. The first model quantified the effect of dark adaptation on sensitivity and had the dark adaptation conditions as a fixed effect (discrete factor). The second was a multivariable model that, along with the dark adaptation, had the BCEA (in log_10_ scale), the BSR (%) and the FP (%) and age as predictors.

Test–retest variability was quantified using the 95% limits of agreement (LoAs) calculated from Bland–Altman plots as the 5th and 95th percentiles of the test–retest differences. The learning effect (practice effect) was quantified as the average difference between the test and the retest. Bland–Altman plots were calculated for both the point-wise data and the average sensitivity. However, the effect of different test metrics on test–retest variability was quantified only with the average sensitivity, because the metrics apply to the whole test.

BSR and the FP are both treated as metrics that could bias the measured sensitivity. Therefore, pairs of tests with a positive difference in average sensitivity (ie, where the second test has higher average sensitivity than the first test) are also expected to have a positive difference in FP and BSR. The opposite is true for pairs with negative differences. This finding was tested using a linear model relating the pair-wise differences in FP and BSR with the pair-wise differences in average sensitivity for all test–retest pairs. The model included random intercepts for the patient. For the BCEA, such directionality was not expected and the absolute pair-wise difference was used instead.

The data extraction and calculation of FP was performed in Matlab (The Mathworks, Natick, MA). All statistical analyses were performed in R (R Foundation for Statistical Computing, Vienna, Austria).

## Results

### Descriptive Statistics

Four of the subjects did not complete the whole series of tests. However, only two were missing the whole retest session. Therefore, we retained all available test–retest pairs from the other 22 subjects, 2 of whom did not perform the retest session for the red stimulus with 30 minutes dark adaptation. In total, we analyzed 66 test–retest pairs for the cyan stimulus and 64 pairs for the red stimulus. The mean (± standard deviation) age of the final sample was 32 ± 10 years and the mean spherical equivalence was −1.22 ± 1.86 diopters. The ART was significantly longer for the cyan stimulus compared with the red stimulus in the 20 minutes and 30 minutes dark adaptation conditions. The estimated differences (± standard error) were 17.7 ± 7.3 ms (*P* = 0.0198) and 19.7 ± 7.5 ms (*P* = 0.009), respectively. We could not detect any statistically significant difference in ART between different dark adaptation times for either the cyan or the red stimulus.

The BCEA, the FP, and the BSR also did not change significantly between dark adaptation conditions and between tests performed with the two stimuli. Descriptive statistics of the tests are reported in Table 1.

### Effect of Eccentricity

The sensitivity with the cyan stimulus was lower at the center and gradually increased towards the periphery (Fig. 1); this pattern was maintained with all dark adaptation conditions. In contrast, the variation in sensitivity at different eccentricities was much smaller with the red stimulus. The difference in sensitivity between red and cyan stimulus was statistically significant in all dark adaptation conditions for the central locations (*P* < 0.05, Bonferroni–Holm correction for six tests).

### Effect of Test Parameters on Sensitivity

**Figure 2.**
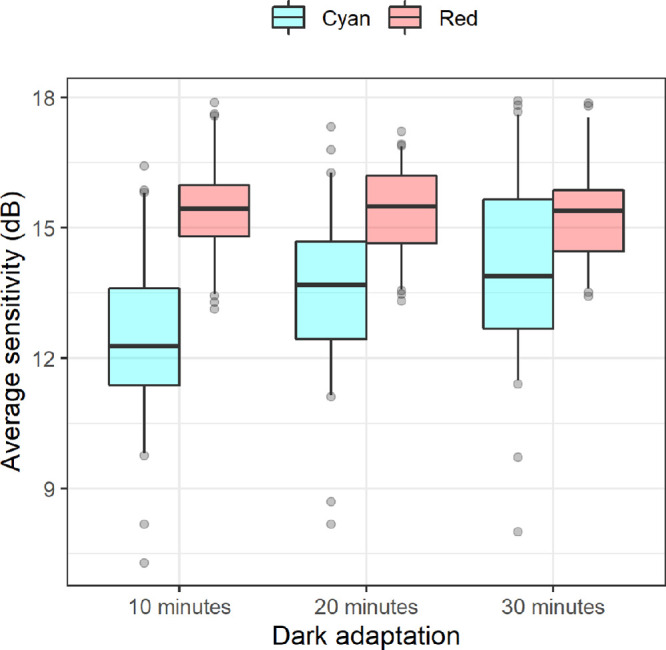
Box plots representing the average sensitivity for tests performed with different dark adaptation protocols. The boxes enclose the 25th to 75th percentiles, the whiskers extend from the 5th to the 95th percentiles.

The average sensitivity (dB) did not change significantly with different dark adaptation conditions for the red stimulus. Conversely, the average sensitivity (dB) did have a significant effect on the sensitivity measured with the cyan stimulus, increasing with longer adaptation times. Average sensitivity values and *P* values are reported in ^1^Table 2 and represented in Figure 2.

**Table 1.** Descriptive Statistics of the Parameters Extracted for the Tests

	Test, Median [Interquartile Range]	
	Cyan	Red
FP responses (%)	3 [1–6]	2 [0–4]
BSR (%)	0 [0–17]	0 [0–17]
BCEA (deg^2^)	1.95 [1.38–3.9]	2.07 [1.32–3.32]
Duration (s)	442 [405–472]	398 [368–431]
Average reaction time (ms)	706 [668–746]	694 [657–734]

BCEA, 95% bivariate contour ellipse area.

**Table 2.** Average Sensitivity Estimates and 95% Confidences Intervals (CIs) for Different Dark Adaptation Conditions (DA)

	Cyan	Red
Average Sensitivity Estimate [95% CIs]		
DA: 10 minutes	12.44 [11.60–13.27]	15.42 [14.96–15.87]
DA: 20 minutes	13.57 [12.73–14.41]	15.37 [14.92–15.83]
DA: 30 minutes	14.16 [13.32–15.00]	15.34 [14.88–15.80]
Pairwise differences		
DA: 10 vs 20 minutes	−1.14 (*P* < 0.0001)	0.05 (*P* = 0.999)
DA: 10 vs 30 minutes	−1.72 (*P* < 0.0001)	0.08 (*P* = 0.999)
DA: 20 vs 30 minutes	−0.59 (p = 0.003)	0.04 (*P* = 0.999)

The bottom part of the table reports the pairwise differences in average sensitivity between conditions. *P* values are corrected for three comparisons using the Bonferroni–Holm method.

In the multivariable model, the FP metric was the only variable demonstrating a significant effect on sensitivity for both the cyan and the red stimuli (Table 3), whereas the BSR metric was only significant for the cyan stimulus (this model also included the dark adaptation condition as a covariate). When tested separately in a model including only each one of the predictors and the dark adaptation condition, the BSR was also significantly positively correlated with sensitivity (*P* < 0.0001 for the cyan stimulus and *P* = 0.004 for the red stimulus).

**Table 3.** Effect of Different Test Parameters on the Average Sensitivity (95% Confidence Intervals)

	Effect on Sensitivity (Estimate [95% CIs])			
	Cyan	*P* Value	Red	*P* Value
log_10_(BCEA) (dB/deg^2^)	−0.057 [0.627 to −0.741]	0.8706	−0.568 [0.016 to −1.152]	0.0589
FPs (dB/%)	0.113 × 10^−2^ [0.158 to 0.068]	<0.0001	0.084 × 10^−2^ [0.123 to 0.044]	0.0001
Blind spot (dB/%)	0.018 × 10^−2^ [0.027 to 0.009]	0.0001	0.009 × 10^−2^ [0.019 to −0.001]	0.0853
Age (dB/year)	−0.026 [0.037 to −0.089]	0.4267	0.003 [0.04 to −0.034]	0.8754

The estimates are derived from multivariable models that included the dark adaptation conditions as fixed effects.

### Test–Retest Variability

**Figure 3.**
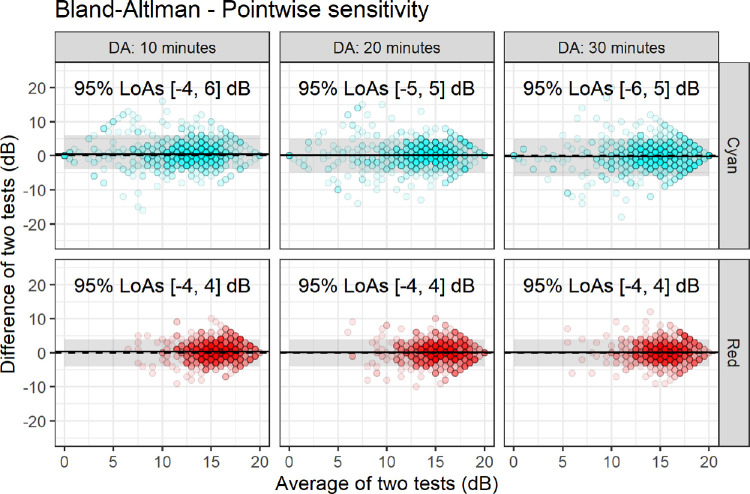
Bland–Altman plots for point-wise test–retest data. The dashed line represents the zero reference level. The solid line represents the average test–retest difference. The shaded areas represent the 95% LoAs.

Bland–Altman plots for point-wise data are reported in Figure 3. The LoAs were very similar between different dark adaptation conditions for both the red and cyan stimuli (also reported in Fig. 3). A ceiling effect is visible for both stimuli. For the cyan stimulus, it is more evident with longer dark adaptation times because of the increase in sensitivity. The cyan stimulus also showed a floor effect, especially for the shortest dark adaptation time. The cyan stimulus had larger LoAs, but also had a wider range of measured values. A significant positive offset of the mean difference was found for the 10 minutes dark adaptation condition for the cyan stimulus (estimate, 0.50 dB; 95% CI, 0.13–0.87 dB; *P* = 0.012).

**Figure 4.**
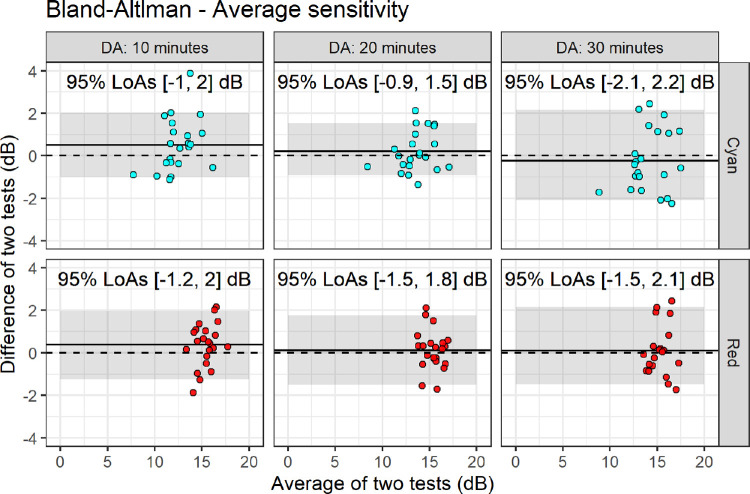
Bland–Altman plots for test–retest data for the average sensitivity. The dashed line represents the zero reference level. The solid line represents the average test–retest difference. The shaded area represents the 95% LOAs.

The LoAs for the average sensitivity were more variable across conditions (Fig. 4), but did not show any systematic change with dark adaptation time for either stimulus. A significant positive offset of the mean difference was found only for the 10 minutes dark adaptation condition for the cyan stimulus (estimate, 0.51 dB; 95% CI, 0.02–0.99 dB; *P* = 0.042).

**Figure 5.**
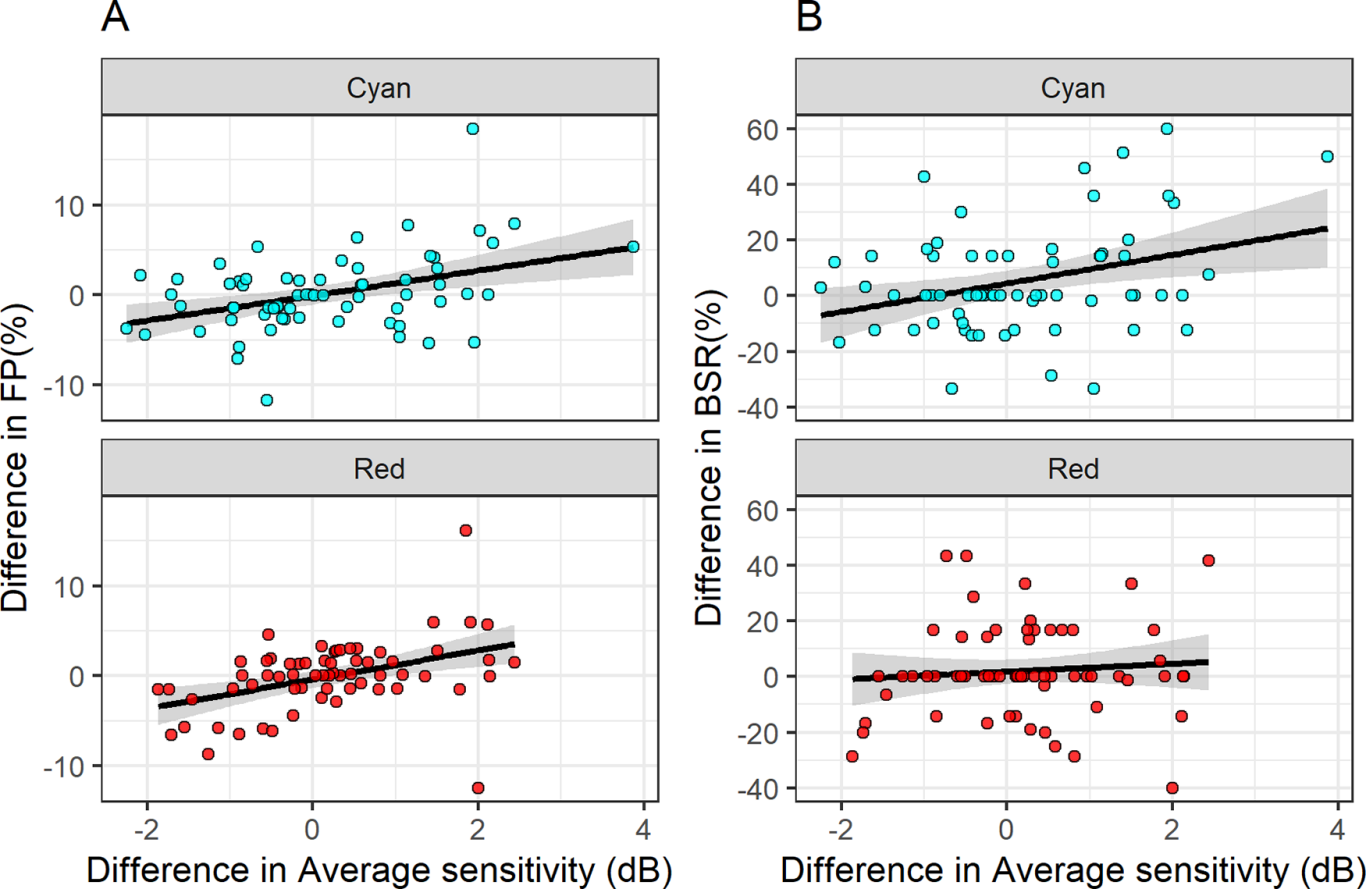
Relationship between the test–retest difference in average sensitivity (horizontal axis) and the differences in reliability indices (vertical axis). (**A**, **B**) The relationship with the test–retest difference in FP reposes and BSR.

The FP test–retest difference was significantly correlated with the test–retest difference of the average sensitivity for both the cyan (*P* = 0.001) and the red stimuli (*P* = 0.003). The slope of the relationship was very similar between the two tests. The overall model was FP_Difference_ = −0.18 + 1.21 × Sensitivity_difference_ (*P* = 0.0001 for the slope and *P* = 0.680 for the intercept). A significant correlation was detected for the BSR with the cyan stimulus (slope = 4.816 %/dB; *P* = 0.012) but not for the red stimulus (*P* = 0.497). Comparing the two models for the cyan stimulus, the R^2^ was larger for the FP (0.132) than the BSR (0.097), reflecting the variability of the two measures. No significant correlation was found for the BCEA (*P* = 0.316 and *P* = 0.591, respectively). The relationships of the test–retest difference in FP and BSR with the test–retest difference in average sensitivity are shown in Figure 5.

## Discussion

We described the effect of different dark adaptation protocols on the sensitivity and test–retest variability on measurements from scotopic two wavelength microperimetry. We also explored how the sensitivity and test–retest variability of these measures could be affected by parameters recorded during the test, including a novel estimate of FP responses using data easily extracted from the device.

Longer periods of dark adaptation significantly increased the sensitivity tested with the cyan stimulus, but not with the red stimulus. This finding aligns with previous knowledge indicating that the response to cyan stimuli, in contrast with the response to red stimuli, is mainly driven by the rod component of the visual response and would therefore be more affected by dark adaptation.^9^^–^^12^ These results are translational because they confirm the importance of using precise dark adaptation protocols to obtain repeatable results, a scenario of more importance when assessing people with AMD, because dark adaptation may be impaired and take longer. In turn, this factor could affect the repeatability of the examination and the ability to discriminate early sensitivity loss.^14^ LoAs for pointwise and average sensitivity were not systematically affected by the dark adaptation protocol. LoAs for average sensitivity with the cyan stimulus were worse (wider) when compared with the red stimulus and were worse with 30 minutes dark adaptation; yet, a systematic increase with longer dark adaptation times was not seen, because they were smaller at 20 minutes compared with 10 minutes. A similar result was observed with the test performed in mesopic conditions.^13^ The test–retest variability was consistently greater for the cyan stimulus, likely reflecting the larger measured dynamic range. Minimal change was observed between the 20 minute and 30 minute dark adaptation conditions. We, therefore, suggest 20 minutes as a practical dark adaptation time for testing protocols. However, tests performed with a larger dynamic range might show greater differences between these two dark adaptation conditions.

Our main results largely align with a previous report investigating test–retest variability for two color S-MAIA in healthy eyes,^9^ where tests were performed after 30 minutes of dark adaptation on a device similar to the one we used, with a 20-dB dynamic range. These investigators reported a similar decrease in sensitivity towards the foveal location for the cyan stimulus, consistent with the decreased density of rod photoreceptors towards the fovea.^15^ Of course, the absorption of the cyan stimulus by the macular pigment could also play a role in this decrease^16^; its effect is, however, difficult to compensate in the absence of optical measurement because it can be very variable among individuals.^17^ The test–retest variability was also larger for the cyan stimulus in the data described by Pfau et al.,^9^ but so was the effective dynamic range of the measurement. They also reported a significantly shorter ART for the red stimulus compared with the cyan. We could partially replicate this observation with the two longest dark adaptation times but the difference was much smaller (19.7 ms for the largest mean difference) than the previous report (96 ms difference between averages). This result is biologically supported by the differences between the rod and cone pathway and was used by Pfau et al.^9^ to justify their findings. In particular, the rod pathway is known to be slower than the cone driven response, with a cone–rod latency of approximately 8 to 20 ms in equal cone and rod dark adaptation conditions.^18^^–^^22^ This finding is compatible with our recorded differences. The rod pathway is also known to decrease its latency when more dark adapted, but we could not find any significant effect of dark adaptation on ART in our dataset; this result could, however, be observed with shorter dark adaption times, which were not tested here. Of course, one limitation of our report and that of Pfau et al.^9^ is that the test was performed with a limited dynamic range. However, later reports measuring test–retest variability with the S-MAIA with an extended dynamic range (36 dB) reported similar test–retest variability.^3^^,^^4^^,^^6^^,^^9^^–^^12^^,^^15^^,^^16^^,^^18^^–^^23^ Our results are therefore generalizable, but ought to be interpreted with caution in light of this technical limitation.

Despite our efforts to minimize the learning (practice) effect through an initial training session, we observed an increase in sensitivity for the cyan stimulus between the two sessions with the shortest dark adaptation session (10 minutes). This condition was more likely to show such an effect, because it was the furthest from the superior limit of the dynamic range and because the test–retest pair included the very first test in the series. A positive offset in the test–retest difference for the same dark adaptation condition was also present for the red stimulus, but it did not reach statistical significance. The limited dynamic range in this case could have masked the learning effect. We also investigated the effect of other test parameters on measured sensitivity and test–retest variability and this is new knowledge. We developed a metric to estimate the probability of FP responses based on data available from the device's XML file and this was more strongly associated with test–retest variability when compared with the BSR. This finding has a potential for translation and clinical usefulness because it could be easily implemented and certainly could be used by researchers on their own data by simply using the calculations we devised for the data in the XML file. In fact, our FP metric was the main determinant of sensitivity when dark adaptation conditions were accounted for and the only predictor that was significant for both the cyan and the red test in the multivariable analysis. Similarly, it was the only statistically significant predictor of test–retest differences for both tests and this finding is particularly noteworthy, considering that the range of sensitivity available for the red stimulus was much smaller. This metric is not entirely novel, based on an idea and the methodology used in standard automated perimetry for more than 20 years.^23^ Until now, the BSR has been used as a metric for false responses to assess the reliability of microperimetric examinations.^3^^,^^6^ This approach, however, has three shortcomings. First, the measure is estimated with poor precision because the blind spot test is only performed approximately once every minute during the examination, resulting in an average of seven trials per examination (interquartile range, 6–7) in our dataset. In contrast, our FP metric is estimated almost continuously throughout the examination. Second, the BSR technique is overly reliant on placement of the optic nerve head landmark and a spurious response can manifest from the stray light of the bright stimuli (10 dB). These disadvantages are recognized in conventional perimetry too and the BSR (known as fixation losses) are increasingly disregarded as a metric of reliability.^24^^,^^25^ Third, the thresholds used to highlight unreliable tests based on BSR are usually derived from values commonly used for conventional perimetry (e.g., 25%).^3^ In conventional perimetry, however, BSR (fixation losses) identify shifts in fixation that would cause the stimulus to fall outside the blind spot. This reasoning obviously does not apply to fundus-tracked perimetry, where eye movements are actively compensated. Our results highlight this weakness in BSR because it was clearly not a significant predictor of test–retest variability and neither was fixation instability.

Our measure of FP yields slightly lower estimates of probability of false responses when compared with the FP metric used in standard automated perimetry, by the Humphrey Field Analyzer (Zeiss Meditec, Dublin, CA) for instance. This is noteworthy.^24^ This could be due to different reasons, including the smaller area of visual field tested and the differences between perimetric tests performed in different adaptation conditions. The MAIA, in contrast with the Humphrey Field Analyzer, for example,^23^ does not record too quick responses as FPs. The lack of a lower limit on the response time (180 ms after stimulus presentation in the Humphrey Field Analyzer) could yield lower estimates of FP. Finally, the predictive power of the equation reported in the results to describe the effect of FP on test–retest variability is very low. This could result from the limited dynamic range of the device. A high FP, for example, could cause a paradoxical decrease in the test–retest variability because the person being assessed would consistently be close to the ceiling value (20 dB) in the measurement. False-negative errors are not tested by the MAIA. Unfortunately, these errors can only be quantified with catch trials that would require modifications to the test procedure.

Finally, the fixation instability measured by the BCEA was not significantly correlated with the test–retest variability; this finding is not unexpected because microperimetry is an examination that uses a fundus tracker to compensate for eye movements. This result needs to be interpreted with caution because visually healthy people do not show areas of sharp changes in sensitivity, such as at the edges of a scotoma, that could be found in patients with AMD. Moreover, spurious correlations between fixation instability and general test performance might exist besides the obvious effect of eye movement, although no such correlation was observed in our data. Additional variability could derive from the fact that a new PRL was determined for each test. However, this factor is unlikely to produce substantial effects in visually healthy people, where the hill of vision is smooth and central fixation is expected to be stable. We performed a more detailed analysis of this aspect, reported in the supplementary material, showing that this was indeed the case.

In conclusion, we show that consistent dark adaptation protocols are essential in determining the repeatability of scotopic microperimetry, especially if the cyan stimulus is used. Moreover, we show that easily accessible data derived from the test can be used to estimate a FP metric, which appears to be a better descriptor of the test performance, and should replace the more commonly used BSR to assess reliability of microperimetric tests.

## Supplementary Material

Supplement 1

## Figures and Tables

**Figure 6.**
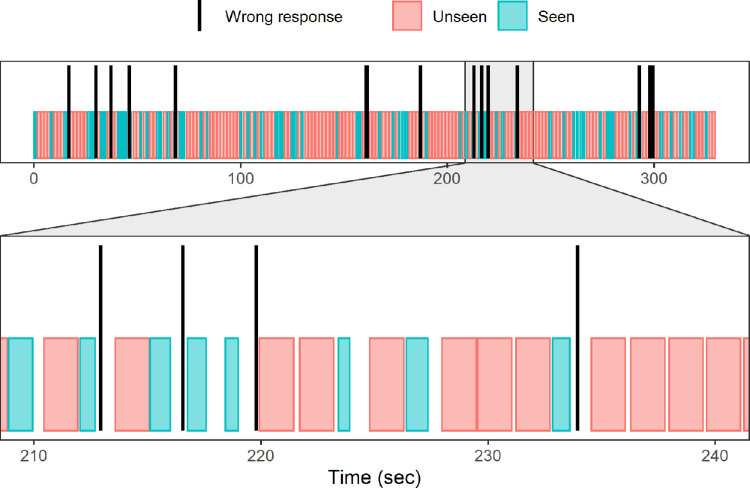
Simulated test sequence based on data from a real test. The colored rectangles indicate the time allocated for seen and unseen stimuli, during which a response was expected or observed. The gaps represent stimulus-free intervals during which no response is expected. The vertical black strokes represent the wrong responses detected during the test.

## Appendix. Calculation of the Rate of FP Responses

MAIA allocates a time window after each stimulus presentation for the patient to respond. The maximum allowed duration of the response window is fixed at 1500 ms. The response window is, however, terminated early, as soon as the patient responds to the stimulus. The actual response times are not reported. However, the intensity of each stimulus presented at each location is recorded and the ART is also reported in the XML file. Knowing that the strategy used is a staircase (2–1 for the scotopic test and 4–2 for the mesopic test), we can derive for which presentations the subject provided a response. For those presentations, we can estimate that the length of the response window was equal to the ART. For the other stimuli, the response window was equal to the entire allowed response time (1500 ms). Finally, we can sum the duration of all these response windows to obtain the time of the test during which a correct response was expected or recorded (Time_cr_).

$$\begin{array}{c} Time_{cr}=N_{seen}\;*\;ART+\;N_{unseen}\;*\;1500\;\mathrm{ms} \end{array}$$

This could then be theoretically subtracted from the total duration of the test to obtain the time during which the wrong pressure events were recorded (Time_wr_). An accurate estimate of the Time_wr_ requires, however, additional adjustments. The test duration recorded in the XML includes the time taken to acquire the initial fundus picture, the PRL assessment and a short training phase of a maximum of eight stimuli. However, WRs are not recorded during this time. The time for the actual test is not reported, but can be estimated. Besides the fixed 1500 ms allocated for unseen stimuli, the MAIA adds a waiting time of 800 ms after every seen stimulus. The average interval allocated for a seen stimulus is then, on average, Interval_seen_ = ART + 800 ms, up to a maximum of 1500 ms. Finally, after all stimuli, a random interval is added, uniformly chosen between 0 and 300 ms (average 150 ms). Therefore, the overall estimated time for the actual test based on the presentations reported in the XML is

$$\begin{array}{ccc} Actual\;test\;time & = & N_{seen}\;*\;\left(Interval_{seen}+\;150\;\mathrm{ms}\right)\\ & & +\;N_{unseen}\;*\;1650\;\mathrm{ms} \end{array}$$

The total Time_wr_ is then calculated as follows

$$\begin{array}{c} Time_{wr}=Actual\;test\;time-Time_{cr} \end{array}$$

Following Olsson et al.,^23^ the rate of FP responses is simply λ = (Number WRs)/Time_wr_ and the probability of a false response within a response window is equal to FP = 1 – e^–(^^λ*1500 ms)^. The FP was reported as a percentage. This is similar to the FP metric calculated by the Humphrey Field Analyzer (Zeiss Meditec, Dublin, CA),^23^ except that the MAIA does not use a lower limit for the response time, meaning that too early responses (<180 ms) are not considered as false responses. A schematic of how the FP responses would appear in a test sequence is reported in Figure 6.
