# Supplementary material for: Determinants of Test Variability in Scotopic Microperimetry: Effects of Dark Adaptation and Test Indices
Source: Transl Vis Sci Technol. 2021 Jan 15;10(1):26. doi: 10.1167/tvst.10.1.26 (PMC7814359; doi:10.1167/tvst.10.1.26)
Supplement: Supplement 1 [file tvst-10-1-26_s001.pdf]

Supplementary material

Distribution of false positive rate

To improve translation of our results, we calculated the FP metric on 813 mesopic tests and 953 scotopic tests from the Northern Ireland Sensory Aging-2 (NISA-2) study [1], consisting of a subset of participants in the NICOLA study [2] which is an ongoing population based study conducted at Queen's University, Belfast. The dataset includes a wide variety of subjects, including healthy participants, and people with diabetes or AMD. The histograms of the distributions are reported in **Figure S1**. The quantiles of the distribution could also be selected as more or less stringent thresholds (reported in **Figure S1**).

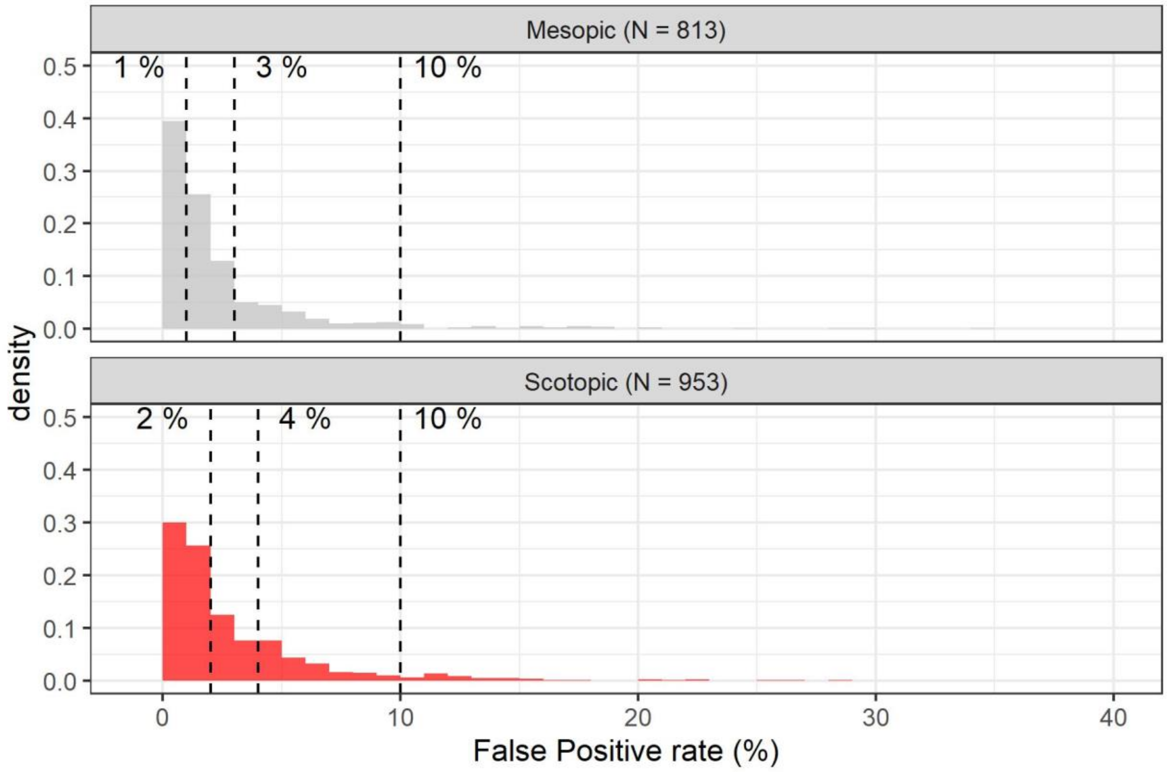

Figure S1. Distribution of FP calculated with the proposed metric in 1766micorperimetric tests. The vertical dashed lines represent the cut-offs at the median, 75th and 95th percentiles (in order from left to right).

Effect of preferred retinal locus position

The Preferred Retinal Locus (PRL) was allowed to vary between test repetitions. To study the effect of PRL position, we aligned all fundus pictures form the same subject with the image from the first test using the RNiftyReg package for R (R Foundation for Statistical Computing, Vienna, Austria). We then used the affine transformation obtained from the alignment process to report all different PRL positions onto the first image. **Figure S2** shows this plot for each subject in the dataset, along with the maximum observed pairwise distance between PRLs (Maximum Distance, MD) and the average distance of the PRLs from their average position (Average Spread, AS).

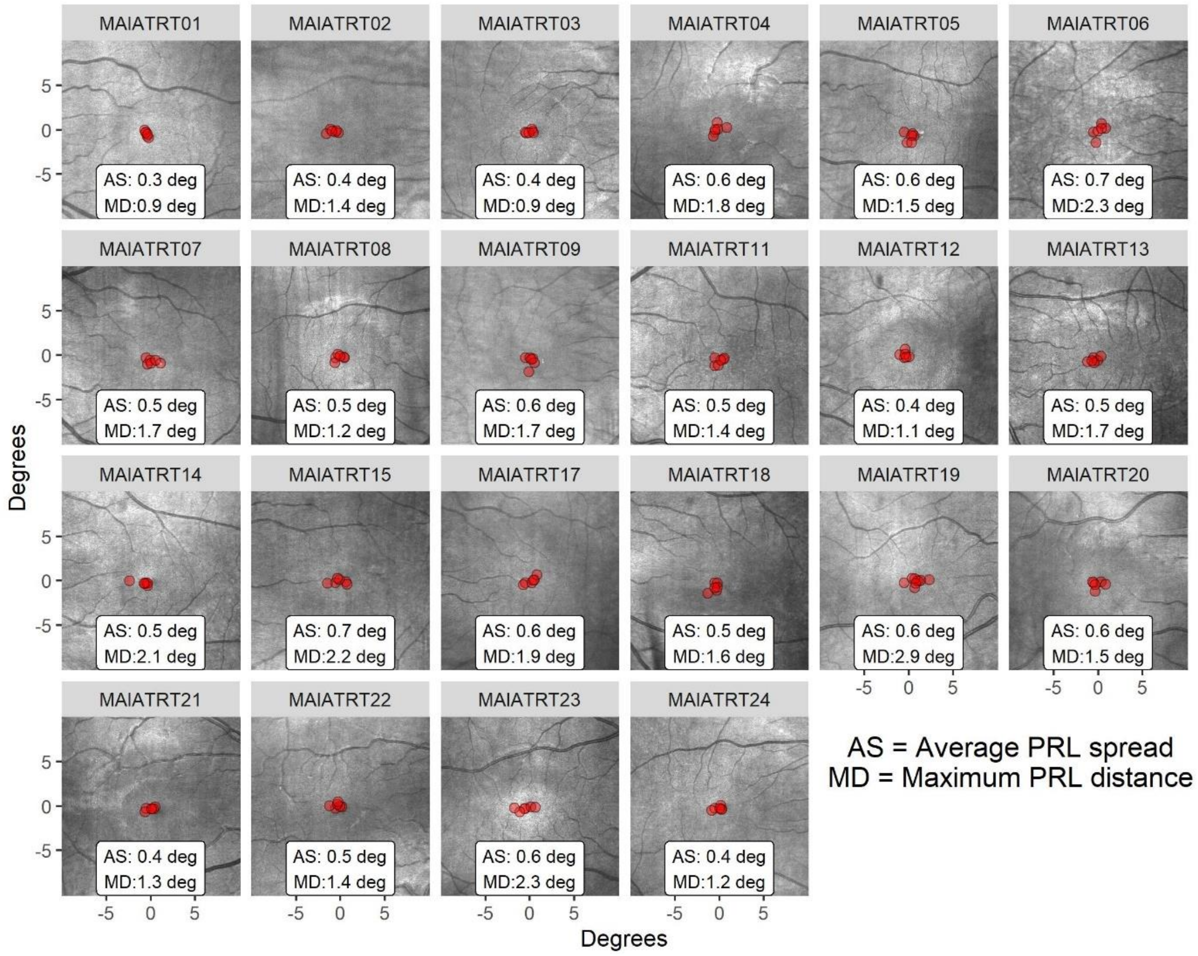

Figure S2. Positions of the Preferred Retinal Locus (PRL) across different tests for each subject. AS = Average Spread; MD = Maximum Distance.

We then studied the effect of the distance between PRLs for each test-retest pair, looking at significant correlations between the PRL distance and the Mean Absolute Difference (MAD)

for test-retest point-wise values (**Figure S3**). We could not find any significant effect for either the Cyan (p = 0.933) or Red scotopic test (p = 0.401).

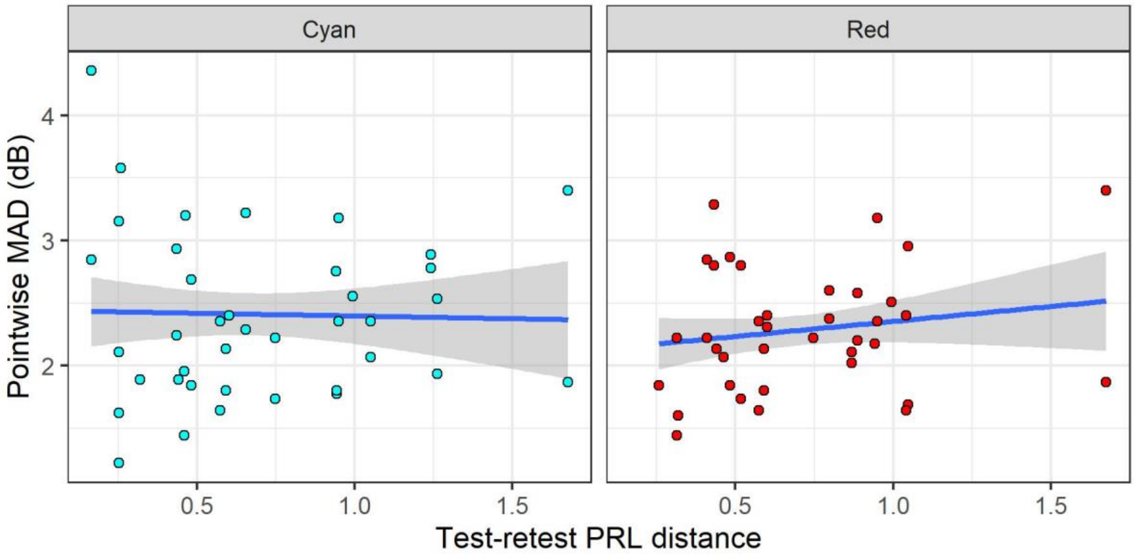

Figure S3. Relationship between test-retest pointwise Mean Absolute Difference (MAD) and Preferred Retinal Locus (PRL) test-retest distance for each test pair.

References

1. The Northern Ireland Sensory Ageing Study - ClinicalTrials.gov. https://clinicaltrials.gov/ct2/show/NCT02788695. Accessed July 24, 2020.
2. NICOLA | Queen's University Belfast. http://www.qub.ac.uk/sites/NICOLA/. Accessed July 24, 2020.
